# BreastGAN: Artificial Intelligence-Enabled Breast Augmentation Simulation

**DOI:** 10.1093/asjof/ojab052

**Published:** 2021-12-11

**Authors:** Christian Chartier, Ayden Watt, Owen Lin, Akash Chandawarkar, James Lee, Elizabeth Hall-Findlay

**Affiliations:** McGill University Faculty of Medicine, Montreal, QC, Canada; Department of Experimental Surgery, McGill University Faculty of Medicine, Montreal, QC, Canada; McGill University, Montreal, QC, Canada; Manhattan Eye, Ear, and Throat Hospital, New York, NY, USA; Division of Plastic and Reconstructive Surgery, McGill University Health Center, Montreal, QC, Canada

## Abstract

**Background:**

Managing patient expectations is important to ensuring patient satisfaction in aesthetic medicine. To this end, computer technology developed to photograph, digitize, and manipulate three-dimensional (3D) objects has been applied to the female breast. However, the systems remain complex, physically cumbersome, and extremely expensive.

**Objectives:**

The authors of the current study wish to introduce the plastic surgery community to BreastGAN, a portable, artificial intelligence (AI)-equipped tool trained on real clinical images to simulate breast augmentation outcomes.

**Methods:**

Charts of all patients who underwent bilateral breast augmentation performed by the senior author were retrieved and analyzed. Frontal before and after images were collected from each patient’s chart, cropped in a standardized fashion, and used to train a neural network designed to manipulate before images to simulate a surgical result. AI-generated frontal after images were then compared with the real surgical results.

**Results:**

Standardizing the evaluation of surgical results is a timeless challenge which persists in the context of AI-synthesized after images. In this study, AI-generated images were comparable to real surgical results.

**Conclusions:**

This study features a portable, cost-effective neural network trained on real clinical images and designed to simulate surgical results following bilateral breast augmentation. Tools trained on a larger dataset of standardized surgical image pairs will be the subject of future studies.

Managing patient expectations is important to ensuring patient satisfaction in aesthetic medicine.^1^ A disconnect between a patient’s preoperative assumption of postoperative result and what is surgically attainable can affect satisfaction and harm the surgeon-patient relationship. In breast surgery, additional technical variables, such as implant type, size, and profile, may further complicate the ability of the patient to imagine a realistic postoperative result. Current popular methods for forecasting surgical results, including arithmetic nomograms and the use of bra sizers, are largely inaccurate.^2,3^ As a result, the most common cause for elective reoperation among breast patients is unsatisfactory implant size.^4,5^ This has amplified plastic surgeons’ responsibility to help patients set realistic surgical goals in the preoperative setting—a longstanding communication challenge in the field.

Patients are more likely to undergo surgery if they have accurate information about the postoperative result.^5^ To this end, computer technology developed to photograph, digitize, and manipulate three-dimensional (3D) objects has been applied to the female breast.^6^ Currently available imaging systems can render a sequence of photographs as a 3D surface and simulate various surgical procedures (breast augmentation, reduction, mastopexy, etc.) to digitally generate a plausible result.^7^ In the years since the first versions of these systems were launched, developers have added features such as automated measurements, breast volume estimates, and breast implant selection.^8^ However, the systems remain complex, physically cumbersome, and extremely expensive, with prices ranging from $12,000 US to $49,000 US (June 2013 prices).^7-10^ Furthermore, they do not incorporate artificial intelligence (AI) technology, instead relying on linear finite elements (computer-generated imagery representing expected soft tissue changes in response to surgery), and may not be based on databases of true, attainable surgical results.^6^

The field of AI is predicated on the rigorous analysis of large datasets for the purpose of making predictions.^11^ Neural networks (NNs) are algorithms designed to replicate decision-making pathways in the human brain. Tasks already mastered by NNs in their current form include image classification, free text analysis, and defeating human experts at abstract strategy games such as chess and “Go.” ^12^ More advanced tasks now being tackled by AI include the generation of “deep fakes,” fake images synthesized from a set of general constraints based on previously learned real images.^13^ These applications require a specific kind of NN, called a generational adversarial network (GAN).^14^ Generative modeling is a subfield of machine learning that concerns itself with automatically recognizing patterns in visual input (training) data with the main goal of generating “fake” examples (images) that are indistinguishable from the training images.^14^ Quintessential examples of GANs include models to turn images of horses into zebras, summer landscapes into winter landscapes, and non-emotive faces into smiling faces. The key to successful GAN development is a careful selection of standardized training images for the algorithm to “learn” from.

While there are few datasets of surgical images sufficiently large for AI studies, plastic surgery “before-and-after” images, primarily meant to be referenced by prospective patients or presented in journals or conferences, provide a large dataset of surgical outcomes. This sets the stage for GAN development on clinical images spanning the entire gamut of plastic surgery procedures (Figure 1).

**Figure 1.**
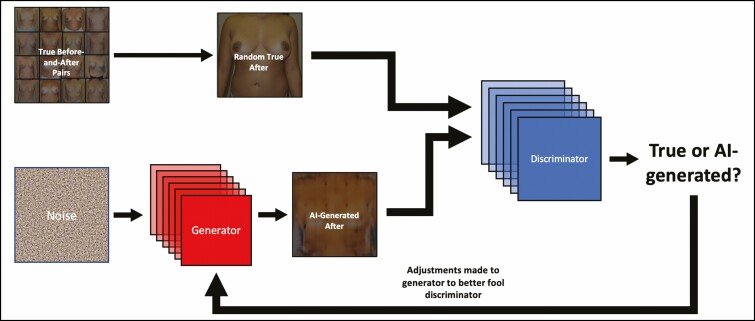
Overview of a clinical generative adversarial network (GAN). AI, artificial intelligence.

The authors of the current study have developed BreastGAN (Montreal, Canada), an AI-driven algorithm trained on real clinical images to automatically simulate breast augmentation outcomes.

## METHODS

### Database Creation

Charts of all patients who underwent bilateral breast augmentation (without mastopexy) performed by the senior author (between January 2003 and January 2018) and who consented to their images being used for research purposes were retrieved and analyzed. Written consent was provided by which the patients agreed to the use and analysis of their data. No intervention was performed on any human or animal patients as part of this study.

Frontal before-and-after images were collected from each patient’s chart, cropped to limit background visualization, centered vertically on the midpoint between the sternal notch and the umbilicus, and centered horizontally on the midpoint between the elbow creases. In total, before-and-after image pairs were collected from 1235 patients and included in the final analysis. The database was split such that 75% (n = 926) of image pairs constituted the “training set,” while 25% (n = 309) of image pairs constituted the “test set.” No features such as implant type, size, or profile were included in the analysis. Features including skin quality/thickness, breast gland density, and level of ptosis only impacted the analysis and results to the extent that they could be observed on the clinical images by the algorithm during training.

### BreastGAN Training

The GAN in this study was developed based on the “pix2pix” framework published by Isola et al and trained on an Nvidia K80 12GB GPU hosted on Google (Alphabet, Mountainview, CA) Colaboratory.^15^ The pix2pix GitHub (GitHub Inc., San Francisco, CA) repository was cloned from https://github.com/junyanz/pytorch-CycleGAN-and-pix2pix.git and mounted in Google Colaboratory, a popular machine learning environment. All images in the database were resized to 850 by 950 pixels for the purpose of training. The training was done up to 250 epochs, or iterations through the entire set of training image pairs. After each epoch, the surgical results generated by the model were retrieved to illustrate the model’s improvement throughout the training process.

### BreastGAN Testing

Testing consisted of introducing the algorithm to the 309 individual frontal before images constituting the test set, recording the outputs (309 AI-generated frontal after images), and comparing the AI-generated frontal after images to the real surgical results.

## RESULTS

### Training and Testing

**Figure 2.**
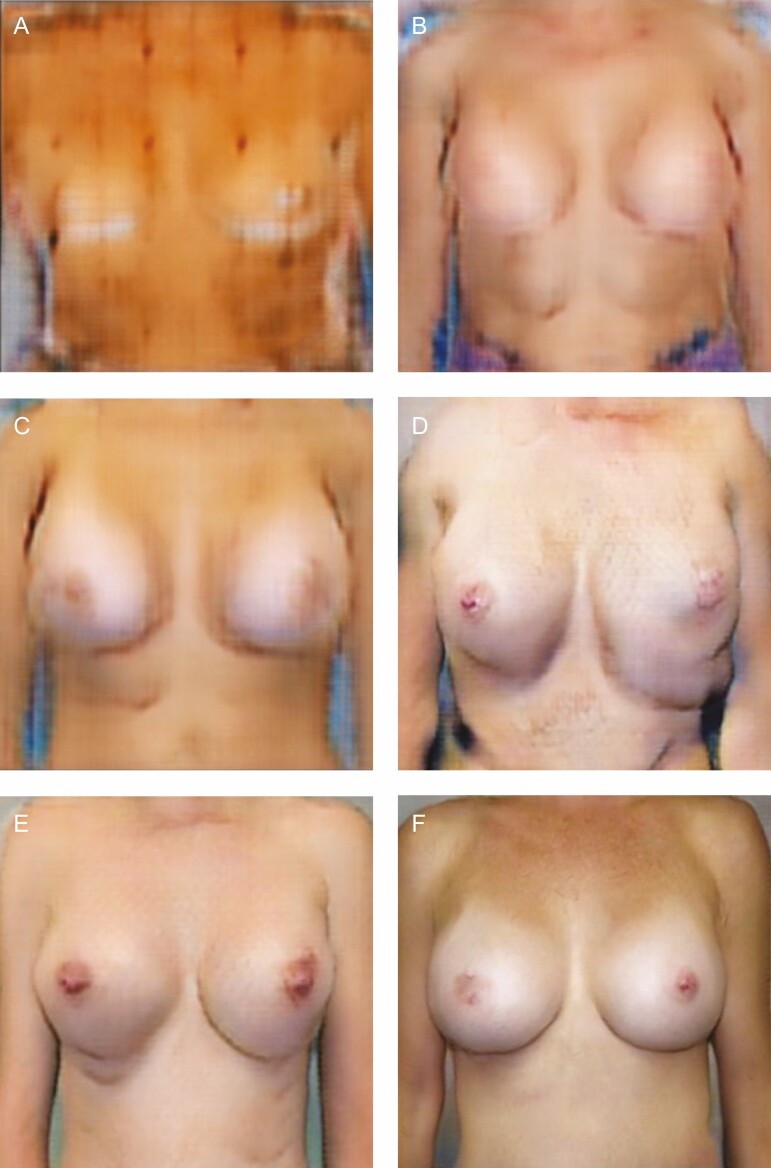
Sample of artificial intelligence-generated surgical results retrieved throughout the training process: (A) Epoch 1, (B) Epoch 50, (C) Epoch 100, (D) Epoch 150, (E) Epoch 200, and (F) Epoch 250.

A representative sample of AI-generated surgical results retrieved from training epochs 1, 50, 100, 200, and 250 are shown in Figure 2.

### Evaluation

**Figure 3.**
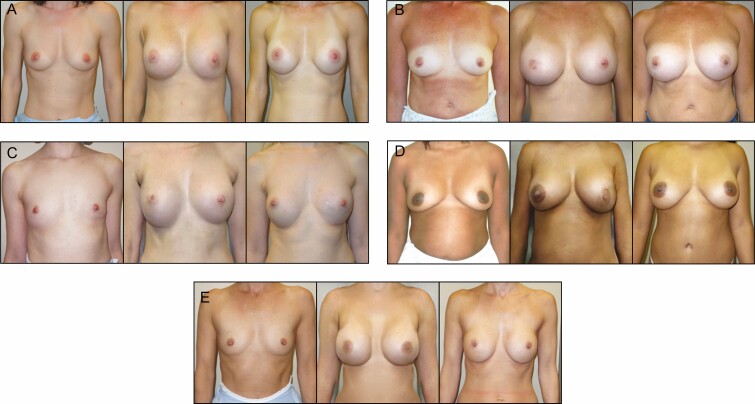
Sample of BreastGAN testing results: (A) 35-year-old female, (B) 42-year-old female, (C) 44-year-old female, (D) 44-year-old female, and (E) 39-year-old female. Leftmost panels are true preoperative images; middle panels are BreastGAN-simulated postoperative results; rightmost panels are true postoperative images.

Standardizing the evaluation of surgical results (test group) is a timeless challenge which persists in the context of GAN-synthesized after images.^16^ While surveys and metrics such as per-pixel mean-squared error exist to compute the geometric distance between AI-generated and authentic after images, we are currently seeking a more holistic approach to the evaluation of surgical images. For now, randomly selected results of GAN testing are shown in Figure 3.

## DISCUSSION

Previous studies have described the use of 3D surface-imaging systems for preoperative planning in plastic surgery.^7,17-20^ They offer a multitude of innovative features to help plastic surgeons better manage their patients’ expectations in the preoperative setting. This shift in the field’s approach to preoperative goal-setting is correlated with increased patient satisfaction.^18^ However, these tools remain too costly for some clinics and medical centers and can only be administered in a clinical setting.

In this study, we propose BreastGAN, an AI-equipped tool capable of simulating breast augmentation from a preoperative image taken. It leverages similar technology as FaceApp (FaceApp Technology Limited, Limassol, Cyprus), an application designed to show users how they would look older, younger, or with different hairstyles, and can be similarly deployed as a mobile application. Consistent with findings published by Isola et al in the index study introducing GAN training on paired images, this study suggests that GANs can be used to accomplish image translation tasks (eg, translate before images into after images) on clinical images.^15^ While BreastGAN in its current form may have fewer features when compared with popular legacy 3D imaging systems, we believe access to our tool will empower patients, allowing them to consider whether to pursue a surgical procedure from the comfort of their own homes.

Readers should be cautioned to interpret this study’s findings considering certain limitations. As will all studies describing the use of AI-equipped tools, model outputs are only as reliable as the data used to train them. In this study, the GAN was trained on surgical images provided by the senior author. This means that each image synthesized by the model will reflect surgical results achieved by one plastic surgeon and not necessarily reproducible by another surgeon. Furthermore, given the relative limitation in available training data (1000 s instead of 10,000 s), the model in its current form has not been designed to output results tailored by implant size, incision type, or photographic angle. This will be the subject of future studies describing GANs trained on a wider array of clinical examples. For the reader’s reference, it has been our experience that suitable results can be achieved with as few as 1000 pairs of training images. To generate surgical results across multiple implant sizes from a single input image, at least 1000 additional pairs of images would be required per additional implant size desired (eg, 1000 pairs of images of patients who received implants less than 250 cc, 1000 pairs between 250 and 500 cc, and 1000 pairs between 500 and 750 cc). It is also worth noting that a GAN trained on images aggregated from multiple surgeons would output the average result achieved by all the contributing surgeons. A version of this tool trained on images provided by other surgeons seeking to implement BreastGAN in their practice would output results consistent with their own surgical results. Furthermore, our results only include simulated breast augmentation in patients who were candidates for breast augmentation as determined by the senior author. Using a neural network to determine which surgery a patient is the best candidate for (augmentation vs augmentation mastopexy, for example) is the subject of an ongoing study. Lastly, as described in the Methods section, features such as skin quality/thickness, breast gland density, and level of ptosis only impacted the analysis and results to the extent that they could be observed on the clinical images by the algorithm during training.

With the lack of a rigorously standardized approach to photographing patients undergoing breast augmentation, the model is limited by the features of the images it was trained on. The training was done on images taken from a wide range of distances/angles and with a wide range of backgrounds. Some before-and-after image pairs were also dissimilar. Subsequent studies will describe a methodology for capturing clinical images optimal for the training of AI tools. Lastly, BreastGAN in its current form has been trained to generate the entire projected postoperative image, not just the augmented breasts. This accounts for distortions in non-breast features including the neck/body contours, hairline, and elbow/armpit creases. Subsequent models may involve generating only the breast to improve the plausibility of the results. Additionally, modifiable variables such as size and implant plane may be added as inputs to provide patients with AI-driven projections to help them make more informed decisions, thereby decreasing revisions and increasing postoperative satisfaction.

### Community-Driven Research

The nature of studies involving the use of GANs—and AI more generally—is that they rely on standardized training data often unavailable in the context of plastic surgery. Thus, the authors of this study call on members of the surgical community to consider the standardization and wider distribution of their data and clinical photographs of consenting patients. This will greatly accelerate the pace of research at the intersection of AI and surgery and make possible the development of more accurate forms of tools such as the one described herein. Furthermore, the authors wish to extend an invitation to their peers interested in developing AI-equipped tools trained on data they have collected to contact our team. The principles underpinning this tool can be applied to other areas of aesthetic surgery, including fillers, neuromodulators, rhinoplasty, facial surgery, and body surgery.

## CONCLUSIONS

The development of preoperative imaging tools capable of simulating breast augmentation has empowered patients by helping them visualize a plausible surgical result. However, these tools remain costly, non-portable, and based on graphical manipulations instead of a database of true surgical results. This has been the impetus for the development of BreastGAN, a generational adversarial network trained on paired before-and-after images of patients undergoing breast augmentation. This tool is portable and can be deployed through a mobile application. GANs trained on a larger dataset of standardized surgical image pairs will be the subject of future studies.
